# Effect of music therapy on anxiety in full-term pregnant women

**DOI:** 10.3389/fpsyt.2024.1429999

**Published:** 2024-09-06

**Authors:** Chao Ji, Juan Li, Qiaole Nie, Shuo Wang

**Affiliations:** ^1^ Qingdao Municipal Hospital, Department of Obstetrics, Qingdao, Shandong, China; ^2^ Beijing Yuedi Music Analgesia Labor Institute, Beijing, China; ^3^ Qingdao Women and Children’s Hospital, Mass Organizations Office, Qingdao, Shandong, China

**Keywords:** music therapy, pregnancy, anxiety, pain, postpartum depression

## Abstract

**Objectives:**

To examine the impact of receptive music therapy on maternal anxiety both during and after the process of childbirth.

**Methods:**

In this experimental study, 217 women were divided into the receptive music therapy and control groups. The first group were exposed to music at intervals of 20 minutes for a duration of 30 minutes during labor. Data were collected using the Pregnant Information Form, the State Anxiety Inventory (STAI), Visual Analogue Scale and Edinburgh postnatal depression scale.

**Results:**

The pregnant women who participated in the music group exhibited reduced scores of STAI, both during the active time (46.42 ± 11.69 vs. 50.21 ± 11.14, 44.37 ± 10.38 vs. 47.56 ± 11.46, P<0.05) and two hours after giving birth(26.32 ± 6.23 vs. 29.55 ± 8.9, 30.38 ± 7.15 vs. 33.08 ± 9.45, P<0.05). At the first stage of labor, pregnant women in the music group experienced dramatically reduced score of pain in active phase (6.39 ± 1.00 vs. 6.91 ± 0.99, P<0.05) and Edinburgh postnatal depression scale at discharged from the hospital (6.68 ± 3.36 vs. 7.66 ± 3.54, P<0.05).

**Conclusion:**

Receptive music therapy is effective in reducing pain during labor and anxiety during prenatal and postnatal periods. The use of receptive music therapy in obstetric care can be an effective tool in preventing anxiety-induced complications.

## Introduction

Anxiety is prevalent during pregnancy, with rates ranging from 18.2% in early pregnancy to 24.6% in late pregnancy. The highest levels of anxiety are observed during late pregnancy (1). As a result of the alterations in physical characteristics and hormonal levels that occur during pregnancy, women are susceptible to experiencing anxiety, which tends to escalate as the pregnancy progresses (2). Pregnancy anxiety can arise from various factors such as physical discomfort, lifestyle adjustments, lack of social support, changes in roles, hormonal and physiological changes, the anticipation of labor and delivery, and experiencing intimate partner violence during and after pregnancy (3).

Women may suffer increased levels of worry and stress when faced with potential complications and health problems throughout pregnancy. Factors such as the lack of clarity over the baby’s health, repeated stays in the hospital, multiple medical examinations and treatments, and a perceived decrease in independence can all contribute to heightened levels of anxiety (4). Research has demonstrated that prenatal stress and maternal psychological problems have a detrimental effect on the growth and development of the fetus, leading to unfavorable outcomes during pregnancy, childbirth, and obstetric procedures (5). This can potentially heighten the likelihood of developing non-communicable diseases, including mental disorders, in the future. Furthermore, when a pregnant woman is exposed to a stressful scenario, her sympathetic nervous system is activated, resulting in an elevated secretion of catecholamines. This leads to increased blood pressure, decreased blood flow to the fetus via the placenta, and can also induce irregular contractions and intensified pain (6). Maternal anxiety and stress during pregnancy have been associated with negative consequences, including premature birth, lower birth weight, postpartum depression, reduced likelihood of breastfeeding, and diminished prenatal bonding (7).

Healthcare professionals should evaluate the levels of anxiety in pregnant women, implement strategies to address it, and direct them towards effective coping mechanisms to promote mental well-being during pregnancy, aiming to decrease the occurrence of mental health issues after childbirth. It is especially important to intervene during the later stages of pregnancy. Employing non-pharmacological relaxation techniques can effectively diminish negative emotions and facilitate a positive birthing encounter for women. Music enhances the physical, emotional, and spiritual well-being of pregnant women and serves as a readily available, affordable, organic, non-intrusive, and non-pharmacological intervention. Receptive music therapy is a therapeutic approach that aims to harmoniously unite the physical, mental, and spiritual aspects of the patient. This study reviews existing literature and presents recent information on the impact of relaxation therapies during pregnancy on maternal mental health issues, as well as pregnancy and delivery outcomes. Evidence suggests interventions throughout the prenatal period can help avoid postpartum depression (8). Music interventions have consistently shown positive effects on mental health and birth weight outcomes, specifically in reducing maternal stress, anxiety, and depression, as well as improving birth weight. These interventions have also been found to result in sustained improvements in maternal physiological indicators during pregnancy and shorter delivery times (9). The objective of this study was to examine the impact of receptive music therapy on maternal anxiety both during and after the process of childbirth.

## Method

**Figure 1:**
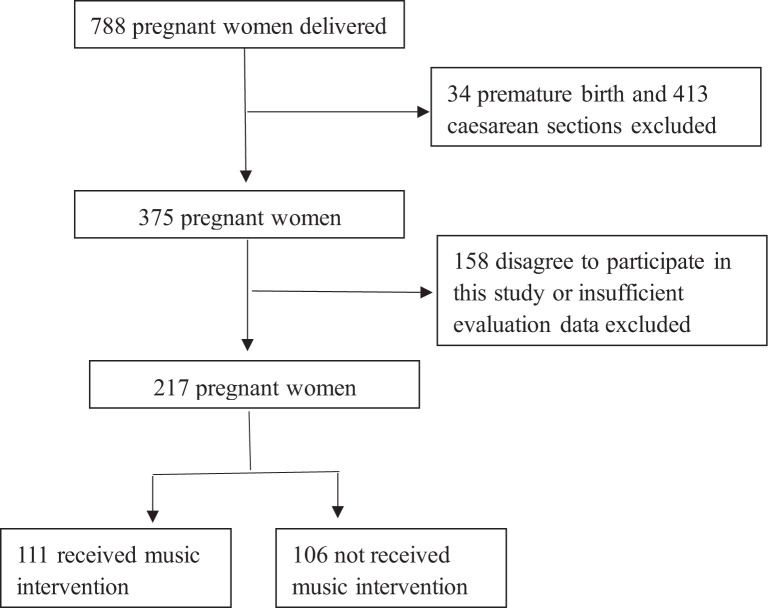
Research protocol.

This study involved the participation of 217 pregnant women who received standard prenatal care. All of them were part of the obstetric population who gave birth at Qingdao Municipal Hospital between March 2023 and August 2023. A total of 111 pregnant women in the music group got the music intervention both throughout the first and second stage of labor. The control group, consisting of 106 individuals, did not undergo any music intervention. The research protocol is presented by **Figure 1**.

### Inclusion criteria

(1) Pregnant women who willingly consented to take part in the research; (2) 18 years old or older; (3) beyond 37 weeks of pregnancy; (4) singleton pregnancy; (5) admitted to the hospital for a minimum of 24 hours; (6) capable of reading, writing, and comprehending; and (7) absence of any cardiovascular illness in the fetus.

### Exclusion criteria

(1) Pregnant women with severe cardiovascular, renal, or neurological conditions or mental disorders such as psychosis, neuroses, or addictions; (2) pregnancies with multiple fetuses; (3) pregnancies with fetal malformations or anomalies, such as congenital disorders; (4) pregnancies with fetal distress that necessitate immediate intervention;(5)cesarean section delivery after vaginal trial labor.

### Music intervention

The music employed in this investigation was characterized by a leisurely tempo that imitated the rhythm of the human heart, specifically 60-75 beats per minute. Additionally, the music had a low to medium pitch and was melodically smooth. Prior to the invention, we engaged in communication with the expectant mothers about their life histories, music tastes and impressive experiences. Researcher helped the pregnant to select the music based on their individual characters and created the respective music repertoires. The adopted musical repertoire comprises classical compositions such as Vivaldi’s The Four Seasons, Schubert’s Lullabies, and traditional Chinese light music like Wang Junxiong’s Music of the Book series. The volume level was regulated within the range of 60-70 decibels. Pregnant women lying on their left side or right side in a dimly lit room to listen to music, in order to minimize the impact of ambient noise. Prior to engaging with music, individuals fine-tuned the loudness of the music to align with their personal tastes. Commencing from the latency period, the pregnant women in the music group were exposed to music at intervals of 20 minutes for a duration of 30 minutes. Music selection and staff training was supervised by a certified music therapist.

### Data collection

The ladies underwent four assessments: latent period, active period, two hours post-delivery, and time of discharge. The assessment tool utilized for the initial three evaluations was the State Anxiety Inventory (STAI), which was established by Spielberger et al. This instrument was employed to quantify the degree of anxiety in expectant mothers. The study utilized the State Anxiety Inventory (STAI-S) and Trait Anxiety Inventory (STAI-T). The evaluation tool used for the most recent examination was the Edinburgh postnatal depression scale (EPDS). The EPDS is a diagnostic instrument specifically developed to assist women in labor in determining whether they are experiencing postpartum depression. It was created by medical professionals from the University of Edinburgh and has been extensively utilized in clinical settings. The assessment has 10 questions that evaluate several facets of the mother’s life, encompassing her emotional state, capabilities, social interactions, and numerous other dimensions. We opted to evaluate postpartum depression using the Edinburgh Postnatal Depression Scale (EPDS) upon discharge from the hospital. The level of pain was assessed using the Visual Analogue Scale. The pain intensity was measured on a scale ranging from 0 to 10, where 0 indicated the lowest level of pain and 10 indicated the maximum level.

The researchers devised a data gathering form to gather personal and obstetric information from medical records. Data for the music and control groups were collected on separate days to mitigate any potential researcher interference between the two groups. During the data collection phase, the pregnant women’s names were documented and their files were appropriately labeled. The researchers ran the investigation until they had obtained the necessary sample size.

### Statistical analysis

The data were analyzed using SPSS (Statistical Package for the Social Sciences) 23.0 software. The χ2 test was employed to create a cross-tabulation of categorical data in order to assess the homogeneity between groups. The mean scores of personal information and assessment of pregnant women were compared using an independent t-test. An acceptable level of significance was considered when p < 0.05.

## Result

A total of 217 female participants were registered in the study over the period from March 2023 to August 2023. All patients successfully finished the research and were included in the analysis. **Table 1** demonstrates that there were no notable disparities in maternal characteristics between the music group and the control group.

**Table 1:** Obstetric history characteristics.

	Age(years)	Gestational week(weeks)	times of pregnancies	Times of deliveries	Body mass index (Kg/m2)	Duration of the first stage of labor	Duration of the second stage of labor	Newborn weight
music therapy group(n=111)	30.95 ± 4.27	39.56 ± 1.16	1.94 ± 1.22	1.33 ± 0.47	27.51 ± 3.42	5.32 ± 3.31	0.85 ± 0.85	3364.37 ± 400.81
control group(n=106)	31.41 ± 4.01	39.41 ± 1.36	1.94 ± 1.00	1.40 ± 0.51	27.72 ± 4.05	5.66 ± 5.29	0.89 ± 0.84	3399.53 ± 433.37
T-value	-0.817	0.922	-0.043	-0.941	-4.16	-0.584	-0.347	-0.621
P-value	0.415	0.358	0.966	0.348	0.678	0.56	0.729	0.535

The STAI scores of two groups were compared and presented in **Tables 2**, **3**. It was evident that pregnant women who participated in the music group exhibited reduced scores of anxiety, both during the active time (46.42 ± 11.69 vs. 50.21 ± 11.14, 44.37 ± 10.38 vs. 47.56 ± 11.46, P<0.05) and two hours after giving birth(26.32 ± 6.23 vs. 29.55 ± 8.9, 30.38 ± 7.15 vs. 33.08 ± 9.45, P<0.05). During the latency period, there was no notable distinction observed between the two groups.

**Table 2:** Comparison of the STAI—S scores between groups.

	latent phase	active phase	2 hours after birth
music therapy group (n=111)	38.01 ± 10.95	46.42 ± 11.69	26.32 ± 6.23
control group (n=106)	39.50 ± 11.72	50.21 ± 11.14	29.55 ± 8.9
T-value	-0.969	-2.439	-3.055
P-value	0.334	0.016*	0.003*

* emphasize the statistical significant.

**Table 3:** Comparison of the STAI—T scores between groups.

	latent phase	active phase	2 hours after birth
music therapy group (n=111)	37.04 ± 8.73	44.37 ± 10.38	30.38 ± 7.15
control group (n=106)	39.31 ± 10.83	47.56 ± 11.46	33.08 ± 9.45
T-value	-1.699	-2.149	-2.362
P-value	0.091	0.033*	0.019*

* emphasize the statistical significant.

Based on **Table 4**, there was no empirical evidence indicating a significant disparity in obstetric complications. We can find the results in **Table 5** that during the initial phase of childbirth, pregnant women in both groups reported similar levels of pain. However, in the active phase, pregnant women in the music group experienced dramatically reduced score of pain (6.39 ± 1.00 vs. 6.91 ± 0.99, P<0.05) and EPDS at discharged from the hospital (6.68 ± 3.36 vs. 7.66 ± 3.54, P<0.05).

**Table 4:** Comparison of the obstetric complications.

	Postpartum hemorrhage	fetal intrauterine distress	meconium staincol amniotic fluid	placental abruption	Precipitate delivery
music therapy group (n=111)	12	3	24	2	18
control group (n=106)	16	5	21	1	20
χ2	0.885	0.620	0.108	0.293	0.264
P-value	0.419	0.491	0.867	1.000	0.721

**Table 5:** Comparison of the pain and EPDS scores.

	pain intensity during the first stage of labor	pain intensity during the second stage of labor	EPDS
music therapy group (n=111)	3.88 ± 1.24	6.39 ± 1.00	6.68 ± 3.36
control group (n=106)	3.94 ± 1.26	6.91 ± 0.99	7.66 ± 3.54
T-value	-0.357	-2.744	-2.103
P-value	0.722	0.007*	0.037*

* emphasize the statistical significant.

## Discussion

Music is utilized in various domains. Listening to music is a cost-effective and natural practice that actively contributes to mental, emotional, and spiritual recovery. Currently, musical interventions are extensively employed in healthcare. Research has demonstrated that receptive music therapy in a serene and soothing setting has led to a reduction in blood pressure and heart rate values among pregnant women (10). Furthermore, they possess a higher level of safety and do not exhibit any notable adverse effects in comparison to pharmaceuticals. Our study revealed that the music helped pregnant women to reduce the level of anxiety throughout the active time and at 2 hours postoperatively. This finding demonstrated the effectiveness of the receptive music therapy at relieving anxiety.

Pregnant women often experience anxiety during labor due to the pain and worry associated with childbirth. This anxiety can result in overall exhaustion, which further intensifies the pain experienced during labor and consequently diminishes the ability to manage the labor process (11). Elevated levels of pain diminish pregnant women’s assurance in the process of labor and childbirth and intensify feelings of unease, so instigating a detrimental loop. Receptive music therapy can offer pain relief during labor and contribute to a positive and gratifying birthing experience for women. Music has the ability to alleviate the stress experienced by pregnant women when they are exposed to painful stimuli. This is achieved by enhancing the release of endorphins, which in turn reduces pain (12). In this study pregnant women in the music group reported a noticeably less intense pain experience in active phase. It is consistent with Amanak’s research (13). Interestingly, the stages of labor pain reduction were found to align with the stages of decreasing STAI scores. It may indicate that the use of music provides pain relief for pregnant women and pain intensity reduction helps to eliminate the additional anxiety caused by the pain.

One study found that women who used receptive music therapy experienced a statistically significant decrease in the duration of the first stage of labor, compared to the control group (14). When a pregnant woman experiences a state of relaxation during labor and delivery, it helps to decrease her anxiety levels and promotes the hormonal processes that are responsible for starting and sustaining labor. Adrenaline, which is released when anxiety is triggered, is a primary suppressor of natural oxytocin and aids in the normal progression of labor and childbirth (15). Our study found no statistically significant disparity in the duration of labor between the two groups. This lack of difference may be attributed to the aggressive obstetric interventions, specifically the administration of intravenous oxytocin throughout labor to heighten the intensity of contractions for pregnant women with weak contractions during labor. The presence of sufficient levels of oxytocin masks the suppressive impact of adrenaline on contractions.

Enhancing maternal welfare during pregnancy facilitates mothers in effectively attending to their own needs during this period, while ensuring better maternal psychological well-being after childbirth contributes to the bond between mother and infant, the quality of child care, and the promotion of exclusive breastfeeding. These factors collectively foster positive growth and development in offspring (16). Antenatal anxiety significantly predicts the occurrence of postpartum depression (17). Receptive music therapy has a beneficial effect on reducing anxiety, which persists even after childbirth (9). The study found that pregnant women in the music group had markedly lower EPDS scores compared to the control group. The findings imply that prenatal acceptance of receptive music therapy can be advantageous in mitigating the occurrence of postpartum depression.

When choosing music, it is important to consider the individual interests of patients. When the music beyond the patient’s range of music propensity was picked from a list provided by the researcher, the results did not show a significant reduction in anxiety (17). One potential reason is that the participants’ favored music may exhibit greater variability in terms of musical attributes such as emotion, intensity, and timbre. This significant variance may have resulted in increased diversity in psychological or physiological effects. The researchers encountered difficulty in selecting music that could adequately represent this diversity, resulting in limited outcomes (18). Music selection and level of familiarity positively influence relaxing. Our study revealed that classical music, preferred music that aligns with a woman’s cultural heritage, and lullabies have the ability to decrease anxiety levels and facilitate the establishment of a strong emotional connection between mother and child, hence enhancing their overall welfare (19). Gentle tunes and expressions conveying affection serve as a reminder to women of their infants. Engaging in a mind-body intervention that involves listening to music while simultaneously stroking the abdomen can effectively alleviate anxiety and enhance prenatal attachment in pregnancies that are considered high-risk. Following the implementation of the lullaby intervention, the average anxiety score reduced by 14.16 points as a result of the music intervention, according to the findings of a study (p < 0.01) (7).

In this study, researchers surveyed pregnant women regarding their musical tastes. The participants were then allowed to listen to their preferred music while being encouraged to gently brush their abdomen. This activity aimed to facilitate communication with their unborn offspring through tactile stimulation and introspection. This behavior fosters prenatal bonding between the mother and fetus, enhancing the mother’s focus on the fetus and facilitating the transmission of affection from the mother to the baby through music and touch. Establishing a tactile and intellectual connection between the mother and the baby will ultimately strengthen the attachment between them (20).

This study presents empirical evidence to corroborate the conclusion that receptive music therapy is efficacious in alleviating anxiety and pain experienced by pregnant women during childbirth. The innovation of this study was the initial evaluation of anxiety levels within the 2-hour period following childbirth. The 2-hour postpartum phase is the subsequent stage after the intrapartum phase and marks the commencement of the postpartum phase. The acquired findings were in line with the scores of STAI during the active phase and EPDS in the postpartum period. Receptive music therapy consistently alleviates anxiety in pregnant women throughout the entire process, starting with labor and extending to the postpartum period. A drawback of this study is the absence of prior introduction and familiarization with receptive music therapy for pregnant women in the music group. Additionally, the implementation of receptive music therapy during delivery was done for the first time. The variability in acceptance and cooperation of receptive music therapy among pregnant women may have influenced the outcomes of the study.

There are two limitations to this study. One is that the study was based on the analysis of data regarding the application of receptive music therapy in the field of obstetric within one hospital. The data collection for evaluation was found to be insufficient due to various factors, including discomfort experienced for pregnant women during active phase. As a result, 152 pregnant women were ultimately excluded from the study after statistical phase, leading to a reduction in the amount of collected data. The other is that music intervention was primarily conducted by nurses. While they have received training and their choice of music has been reviewed by a music therapist, it is important to acknowledge that they may lack sufficient professional knowledge in music therapy and their level of expertise may vary. Because of the different abilities of the conductor performing the music therapy, this study may have led to inconsistent results in terms of anxiety and pain improvement compared to other studies.

## Conclusion

There is a need to focus on the mental health of women during pregnancy as the emotional perception of the mother affects the fetus on an emotional, affective and organic level. Receptive music therapy is an inexpensive, effective and easy to use application. Receptive music therapy is effective in reducing pain during labor and anxiety during prenatal and postnatal periods. The use of receptive music therapy in obstetric care can be an effective tool in preventing anxiety-induced complications. Healthcare professionals can incorporate this musical intervention into the psychosocial care of pregnant women as a complementary and supportive practice.

## Data Availability

The raw data supporting the conclusions of this article will be made available by the authors, without undue reservation.
